# Supplementary material for: Skin Manifestations in COVID-19: Prevalence and Relationship with Disease Severity
Source: J Clin Med. 2020 Oct 12;9(10):3261. doi: 10.3390/jcm9103261 (PMC7599735; doi:10.3390/jcm9103261)
Supplement: Supplementary file 1 [file jcm-09-03261-s001.pdf]

Supplementary Material

Summary of contents

1. Supplementary Table 1 p 2 - 8

1. Selected case studies:

- Suppl. Figure 1 A and B p 9
- Suppl. Figure 2 A and B p 10
- Suppl. Figure 3 A and B p 11
- Suppl. Figure 4 A and B p 12

Table S1. Case-by-case description of the 58 patients included in the study.

	Sex	Age	Cutaneous presentation	COVID-19 symptoms other than cutaneous	Days from the start of COVID-19 to the appearance of skin lesions	Covid-19 PCR	Covid-19 serology	Chest X-ray	CPR mg/dL	Lymphocytes cell/mm3	Ferritin ng/ml	LDH mg/dL	D-dimer ng/ml	Histological pattern H/E	DIF	AntiC9 IHQ	Treatment	Hospitalization required	ICU required
1	Male	23	Chilblain-like		-	Negative	Negative	Normal	0.40	1200	936	190	300	Chilblain-like pattern	Lichenoid + vasculopathic	Positive		No	No
2	Male	42	Chilblain-like		-	Negative	Negative	-	0.40	2000	197	153	200	Chilblain-like pattern	Lichenoid + vasculopathic	Positive		No	No
3	Male	47	Chilblain-like	Cough	4	Negative	Negative	-	0.40	1100	198	193	200	-	-	-		No	No
4	Female	26	Chilblain-like	Anosmia, Augesia, Fever	21	Negative	Positive	-	0.40	1800	14	156	300	-	-	-		No	No
5	Male	26	Chilblain-like		-	Negative	Negative	-	0.40	1200	106	137	200	Chilblain-like pattern	Lichenoid + vasculopathic	Positive		No	No
6	Female	25	Chilblain-like		-	-	Negative	-	0.40	1400	67	174	200	-	-	-		No	No
7	Male	47	Chilblain-like	Anosmia, Augesia, Cough, Diarrhoea, Fever	30	Positive	-	-	0.40	1900	143	197	200	Chilblain-like pattern	Vasculopathic	Positive		No	No

8	Male	26	Chilblain-like	Cough	0	Negative	Positive	Normal	0.40	2000	114	175	300	Chilblain-like pattern	Vasculopathic	Positive		No	No
9	Female	54	Chilblain-like	Myalgias	0	Negative	Negative	Normal	0.40	1600	69	217	200	Chilblain-like pattern	Negative	Positive		No	No
10	Male	55	Chilblain-like	Fever	17	-	Positive	-	0.62	2500	868	179	6000	Chilblain-like pattern	-	Negative		Yes	No
	**Sex**	**Age**	**Cutaneous presentation**	**COVID-19 symptoms other than cutaneous**	**Days from the start of COVID-19 to the appearance of skin lesions**	**Covid-19 PCR**	**Covid-19 serology**	**Chest X-ray**	**CPR mg/dL**	**Lymphocytes cell/mm3**	**Ferritin ng/ml**	**LDH mg/dL**	**D-dimer ng/ml**	**Histological pattern H/E**	**DIF**	**AntiC9 IHQ**	**Treatment**	**Hospitalization required**	**ICU required**
11	Male	16	Chilblain-like		-	Negative	Negative	-	0.40	3000	47	179	200	Chilblain-like pattern	Vasculopathic	Positive		No	No
12	Male	50	Chilblain-like	Cough, Dyspnea, Fever	34	Positive	-	Bilateral interstitial pattern	30.40	500	1020	316	10000	-	-	-	HQ, L/R, CO, TC	Yes	Yes
13	Female	29	Chilblain-like		-	-	Negative	-	0.40	1800	4	167	300	Chilblain-like pattern	Negative	Positive		No	No
14	Female	36	Chilblain-like	Asthenia, Cough	17	Positive	Negative	Bilateral interstitial pattern	0.40	3000	19	172	200	Psoriasiform	Negative	-		No	No
15	Female	16	Chilblain-like		-	Negative	Negative	-	0.40	1200	5	141	200	Chilblain-like pattern	Vasculopathic	Positive		No	No
16	Male	17	Chilblain-like		-	Negative	Negative	-	0.40	1900	67	189	200	Chilblain-like pattern	Negative	Positive		No	No

17	Female	52	Chilblain-like	Cough	4	-	Positive	-	-	-	-	-	-	-	-	-		No	No
18	Female	76	Generarized maculo-papular eruption	Diarrhoea, Fever	20	Positive	-	Bilateral interstitial pattern	4.50	800	598	328	600	-	-	-	HQ, AZT, L/R, CO, STX	Yes	Yes
19	Female	80	Generarized maculo-papular eruption	Cough, Diarrhoea, Fever, Headache	14	Positive	-	Bilateral interstitial pattern	2.54	600	281	243	200	Spongiotic dermatitis	-	Negative	HQ, AZT, L/R, CO	Yes	No
20	Female	50	Generarized maculo-papular eruption	Anosmia, Augesia, Cough, Diarrhoea, Dyspnea, Fever, Myalgias	4	Positive	-	Bilateral interstitial pattern	6.66	600	112	288	400	Interface + spongiotic dermatitis	-	Negative	HQ, AZT, L/R	Yes	No
	**Sex**	**Age**	**Cutaneous presentation**	**COVID-19 symptoms other than cutaneous**	**Days from the start of COVID-19 to the appearance of skin lesions**	**Covid-19 PCR**	**Covid-19 serology**	**Chest X-ray**	**CPR mg/dL**	**Lymphocytes cell/mm3**	**Ferritin ng/ml**	**LDH mg/dL**	**D-dimer ng/ml**	**Histological pattern H/E**	**DIF**	**AntiC9 IHQ**	**Treatment**	**Hospitalization required**	**ICU required**
21	Female	70	Generarized maculo-papular eruption	Fever, Myalgias	0	Negative	Positive	Normal	3.00	600	244	264	1600	Interface + spongiotic dermatitis	Negative	Negative		No	No
22	Female	87	Generarized maculo-papular eruption	Dyspnea, Fever, Myalgias	16	Positive	-	Bilateral interstitial pattern	5.67	600	341	478	1500	Spongiotic dermatitis	-	Negative	HQ, AZT	Yes	No

23	Male	51	Generarized maculo-papular eruption	Cough, Dyspnea, Fever, Headache	11	Positive	-	Bilateral interstitial pattern	14.91	1100	1406	239	500	Spongiotic dermatitis	-	Negative	HQ, AZT, L/R, Rem	Yes	No
24	Male	58	Generarized maculo-papular eruption	Asthenia, Fever	20	Positive	Positive	Bilateral interstitial pattern	11.32	1400	212	234	600	Subcorneal pustulosis	Negative	-	HQ, AZT, L/R, CO	Yes	No
25	Male	57	Generarized maculo-papular eruption	Cough, Diarrhoea, Dyspnea, Fever, Myalgias	20	Positive	-	Bilateral interstitial pattern	3.14	800	356	212	200	Urticariform	-	Negative	HQ, AZT, L/R, CO, TC	Yes	Yes
26	Male	64	Generarized maculo-papular eruption	Augesia, Cough, Fever	26	Positive	-	Bilateral interstitial pattern	14.95	800	693	322	300	Interface + spongiotic dermatitis	-	Negative	HQ, AZT, L/R, TC	Yes	No
27	Female	64	Generarized maculo-papular eruption	Asthenia, Augesia, Fever, Myalgias	3	Negative	Negative	-	3.37	700	175	242	4400	Interface + spongiotic dermatitis	Negative	Positive		No	No
28	Male	40	Generarized maculo-papular eruption	Cough, Diarrhoea, Fever, Myalgias	24	Negative	-	Bilateral interstitial pattern	31.55	900	1031	519	600	Spongiotic dermatitis	-	Negative	HQ, AZT, L/R, CO, TC, ANK	Yes	Yes
	**Sex**	**Age**	**Cutaneous presentation**	**COVID-19 symptoms other than cutaneous**	**Days from the start of COVID-19 to the appearance of skin lesions**	**Covid-19 PCR**	**Covid-19 serology**	**Chest X-ray**	**CPR mg/dL**	**Lymphocytes cell/mm3**	**Ferritin ng/ml**	**LDH mg/dL**	**D-dimer ng/ml**	**Histological pattern H/E**	**DIF**	**AntiC9 IHQ**	**Treatment**	**Hospitalization required**	**ICU required**

29	Male	25	Generarized maculo-papular eruption	Cough, Dyspnea, Fever	34	Positive	-	Bilateral interstitial pattern	-	-	-	-	-	Other	-	Negative	HQ, AZT, L/R, CO, TC	Yes	Yes
30	Male	78	**G**rover's disease and other papulo-vesicular eruptions[1]	Asthenia, Augesia, Cough, Dyspnea, Vomit	22	-	-	Bilateral interstitial pattern	8.65	1000	636	291	5200	Grove's disease pattern	Negative	Negative	HQ, AZT, L/R	Yes	No
31	Male	61	**G**rover's disease and other papulo-vesicular eruptions[1]	Asthenia, Cough, Diarrhoea, Fever, Myalgias	39	Positive	-	Bilateral interstitial pattern	10.46	600	764	662	10000	Grove's disease pattern	-	Positive	HQ, AZT, L/R, CO, TC, ANK	Yes	Yes
32	Female	50	**G**rover's disease and other papulo-vesicular eruptions[1]	Asthenia, Augesia, Cough, Dyspnea, Fever	10	Positive	-	Bilateral interstitial pattern	4.96	500	26	285	800	Grove's disease pattern	Negative	Negative	HQ, AZT, L/R, Rem	Yes	No
33	Female	24	**G**rover's disease and other papulo-vesicular eruptions[2]	Cough, Dyspnea	0	Negative	Positive	Other pattern	5.60	1200	-	187	700	-	-	-	CO	No	No
34	Male	38	**G**rover's disease and other papulo-vesicular eruptions[2]	Anosmia, Augesia, Cough, Fever	14	Negative	Positive	Normal	1.00	800	159	239	900	Interface dermatitis	Negative	-		No	No

	Sex	Age	Cutaneous presentation	COVID-19 symptoms other than cutaneous	Days from the start of COVID-19 to the appearance of skin lesions	Covid-19 PCR	Covid-19 serology	Chest X-ray	CPR mg/dL	Lymphocytes cell/mm3	Ferritin ng/ml	LDH mg/dL	D-dimer ng/ml	Histological pattern H/E	DIF	AntiC9 IHQ	Treatment	Hospitalization required	ICU required
35	Female	66	Grover's disease and other papulo-vesicular eruptions[3]	Cough, Fever	4	-	-	Bilateral interstitial pattern	18.15	1200	632	354	400	-	-	-	HQ, AZT, L/R, CO, TC	Yes	Yes
36	Female	36	Grover's disease and other papulo-vesicular eruptions[4]	Anosmia, Diarrhoea	9	Positive	-	Bilateral interstitial pattern	0.40	1700	70	182	500	Folliculitis	Negative	-	HQ, AZT	Yes	No
37	Male	46	Grover's disease and other papulo-vesicular eruptions[4]	Cough, Dyspnea, Fever	11	Positive	-	Bilateral interstitial pattern	6.09	700	684	509	600	Folliculitis	-	-	HQ, AZT, L/R, CO	Yes	Yes
38	Female	78	Urticarial eruption	Asthenia, Cough, Fever, Myalgias, Vomit	28	Positive	-	Bilateral interstitial pattern	5.88	1300	903	289	8100	Interface + spongiotic dermatitis	Negative	Negative	HQ, AZT, CO, Rem, STX	Yes	Yes
39	Female	57	Urticarial eruption	Asthenia, Cough, Diarrhoea, Dyspnea,	43	Positive	-	Bilateral interstitial pattern	3.94	1000	776	389	900	Spongiotic dermatitis	Negative	Negative	HQ, AZT, L/R, CO, TC	Yes	Yes

	Sex	Age	Cutaneous presentation	COVID-19 symptoms other than cutaneous	Days from the start of COVID-19 to the appearance of skin lesions	Covid-19 PCR	Covid-19 serology	Chest X-ray	CPR mg/dL	Lymphocytes cell/mm3	Ferritin ng/ml	LDH mg/dL	D-dimer ng/ml	Histological pattern H/E	DIF	AntiC9 IHQ	Treatment	Hospitalization required	ICU required
				Fever, Myalgias															
40	Male	79	Urticarial eruption	Asthenia, Dyspnea, Fever, Myalgias	28	Negative	Positive	Bilateral interstitial pattern	-	-	-	-	-	Spongiotic dermatitis	Negative	Negative	HQ, AZT, L/R, CO	Yes	Yes
41	Female	35	Urticarial eruption	Anosmia, Asthenia, Augesia, Diarrhoea, Fever, Headache	8	Positive	-	Normal	-	-	-	-	-	Urticariform	-	Negative	HQ, AZT, L/R	Yes	No
42	Female	80	Livedo reticularis	Cough, Diarrhoea, Dyspnea	4	Positive	-	Bilateral interstitial pattern	15.43	500	795	415	10000	Thrombotic microangiopathy	Vasculopathic	Positive	HQ, AZT, L/R, CO	Yes	No
43	Male	47	Livedo reticularis	Cough, Fever	17	Positive	-	Bilateral interstitial pattern	9.78	500	1199	242	1300	Spongiotic dermatitis	Negative	Negative	HQ, AZT, L/R, CO	Yes	No
44	Male	71	Livedo reticularis	Cough, Dyspnea, Fever	29	Positive	-	Bilateral interstitial pattern	18.16	700	3244	384	700	Interface + spongiotic dermatitis	Negative	Positive	HQ, AZT, L/R, CO, TC, Rem, ANK	Yes	Yes

45	Male	74	Livedo reticularis	Dyspnea, Fever	31	-	Positive	Bilateral interstitial pattern	-	-	-	-	-	Spongiotic dermatitis	Negative	Negative	HQ, AZT, L/R, CO	Yes	Yes
46	Male	69	Others: Pressure-induced ischemic necrosis in prolonged coma patient	Asthenia, Dyspnea, Fever, Myalgias	19	Positive	-	Bilateral interstitial pattern	30.78	800	1624	508	2000	Other	Vasculopathic	Positive	HQ, AZT, L/R, CO, TC, ANK	Yes	Yes
47	Male	63	Others: Hematoma	Asthenia, Cough, Dyspnea, Fever, Myalgias	8	Negative	-	Bilateral interstitial pattern	1.53	600	373	4094	3200	-	-	-	HQ, AZT	Yes	No
48	Male	45	Others: Lichen planus	Anosmia, Asthenia, Augesia, Fever, Headache	29	Negative	Positive	-	0.40	1200	233	246	500	Interface dermatitis	Lichenoid	-		No	No
	**Sex**	**Age**	**Cutaneous presentation**	**COVID-19 symptoms other than cutaneous**	**Days from the start of COVID-19 to the appearance of skin lesions**	**Covid-19 PCR**	**Covid-19 serology**	**Chest X-ray**	**CPR mg/dL**	**Lymphocytes cell/mm3**	**Ferritin ng/ml**	**LDH mg/dL**	**D-dimer ng/ml**	**Histological pattern H/E**	**DIF**	**AntiC9 IHQ**	**Treatment**	**Hospitalization required**	**ICU required**
49	Male	78	Others: Contact dermatitis	Dyspnea, Fever	33	Positive	-	Bilateral interstitial pattern	36.98	600	525	417	800	-	-	-	HQ, AZT, L/R, CO, TC	Yes	Yes

50	Male	68	Others: Psoriasis	Asthenia, Cough, Dyspnea, Fever, Myalgias	37	-	Positive	Unilateral interstitial pattern	28.16	500	2628	457	1000	Subcorneal pustulosis	Negative	-	HQ, AZT, L/R, CO, TC	Yes	No
51	Female	92	Others: Generalized fixed drug eruption	Dyspnea	-6	Negative	Positive	Unilateral interstitial pattern	1.11	1500	38	316	2100	Other	Lichenoid	-		Yes	No
52	Female	74	Others: Contact dermatitis	Asthenia, Cough, Dyspnea, Fever	34	Positive	-	Bilateral interstitial pattern	0.40	600	73	322	800	-	-	-	HQ, AZT, L/R, CO, TC	Yes	Yes
53	Female	58	Others: Benign familial pemphigus	Cough, Diarrhoea, Fever, Vomit	8	Positive	-	Normal	6.17	800	558	295	400	-	-	-	HQ, AZT, L/R	Yes	No
54	Male	61	Others: Pressure-induced ischemic necrosis in prolonged coma patient	Cough, Fever	37	Positive	-	Bilateral interstitial pattern	15.51	600	635	293	800	-	-	-	HQ, AZT, CO, TC, Rem	Yes	Yes
55	Female	46	Others: Chronic graft-versus-host disease	Asthenia, Diarrhoea, Fever, Myalgias	1	Negative	-	Bilateral interstitial pattern	0.44	1600	1006	163	1100	-	-	-	HQ	Yes	No
**Sex**	**Age**	**Cutaneous pre**	**COVID-19 symptoms other than cutaneous**	**Days from the start of COVID-**	**Covid-19 PCR**	**Covid-19 serology**	**Chest X-ray**	**CPR mg/dL**	**Lymphocytes**	**Ferritin ng/ml**	**LDH mg/dL**	**D-dimer ng/ml**	**Histological patte**	**DIF**	**AntiC9 IHQ**	**Treatment**	**Hospitalization required**	**ICU required**	**Sex**

		sen tati on		19 to the appearan ce of skin lesions					cell/ mm 3				rn H/E						
56	Fem ale	72	Others: Stasis dermatitis	Cough, Dyspnea	11	-	-	Other pattern	2.01	1300	231	299	400	Other	Vasculop athic	-	HQ, AZT, L/R, CO	Yes	No
57	Fem ale	59	Others: Dermatoph ytosis	Dyspnea, Fever	26	Positive	-	Bilateral interstiti al pattern	4.84	1600	912	503	9900	-	-	-	HQ, AZT, L/R, TC	Yes	Yes
58	Male	69	Others: Eruptive cherry angiomas	Cough, Dyspnea, Fever	17	Positive	-	Bilateral interstiti al pattern	9.09	300	2195	327	3400	-	-	-	HQ, AZT, L/R, CO, ANK	Yes	Yes

Grover's disease and other papulo-vesicular eruptions: [1]: Grover disease; [2]: chickenpox; [3]: herpes zoster; [4]: *Pityrosporum spp* folliculitis. HQ: Hydroxychloroquine; AZT: Azithromycin; L/R: Lopinavir/ritonavir; TC: Tocilizumab; Rem: Remdesivir; CO: Systemic corticosteroids; ANK: Anakinra; STX: Siltuximab.

Supplementary Figures

Selected Case Studies

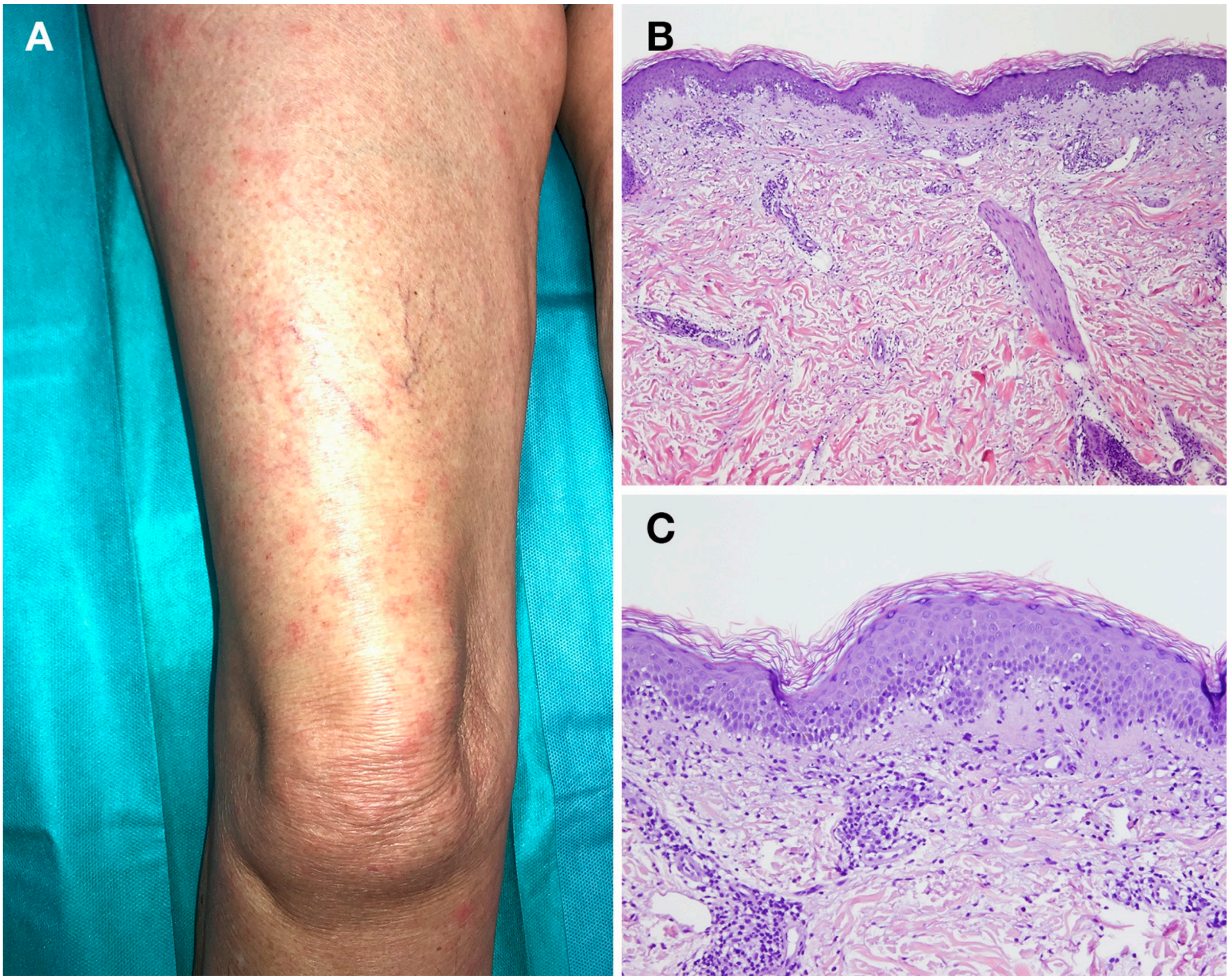

Figure S1. Patient 21 from Supplementary Table 1. (A) 70-year-old woman presented erythematous macules that affected her lower extremities, associated with fever and dry cough. COVID-19 PCR was negative, but IgG serology was positive on follow-up; (B) A skin biopsy performed on the thigh showed an interface dermatitis with perivascular inflammatory infiltrates in the superficial dermis (Hematoxylin-eosin stain, original magnification x40). Direct immunofluorescence was negative.

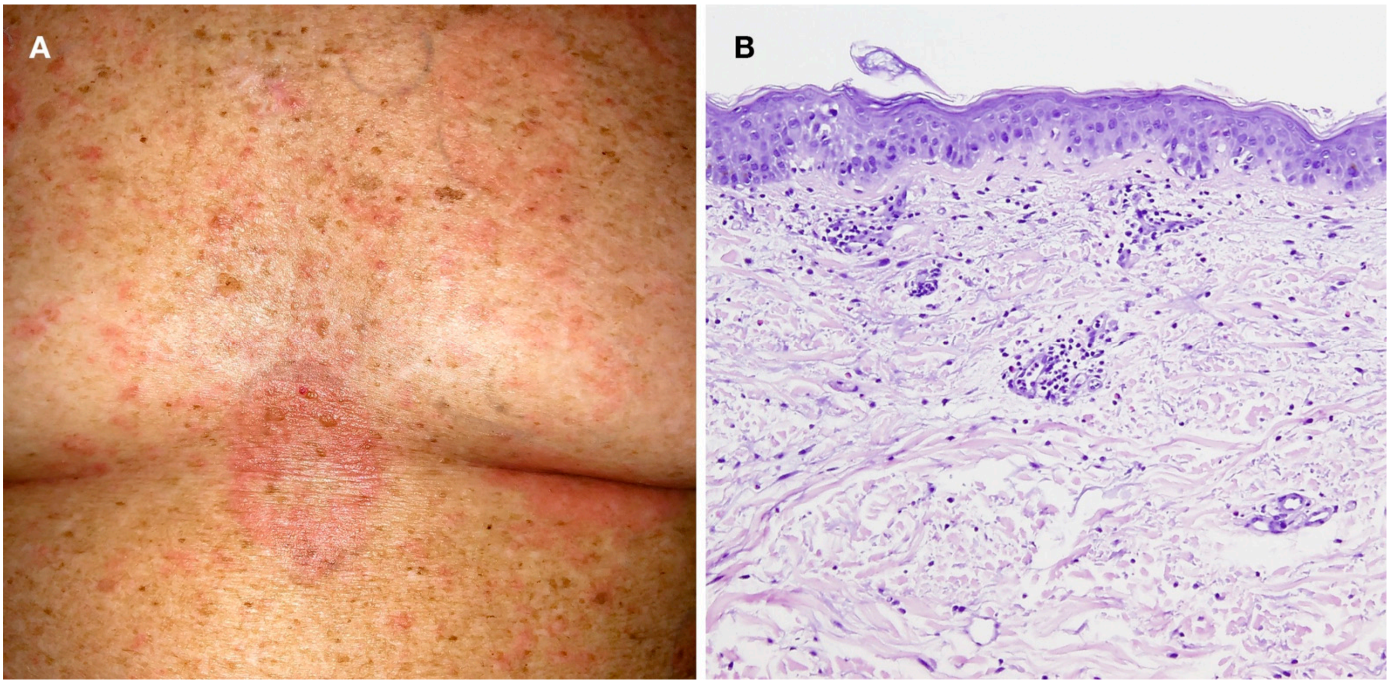

Figure S2. Patient 35 from Supplementary Table 1**.** (A). 77-year-old woman with confirmed diagnosis of COVID-19. Erythematous papules and plaques on the chest and upper abdomen on the 21th day from diagnosis; (B). Mild interface dermatitis and perivascular inflammatory lymphocytic infiltrates (with scarce eosinophils). Hematoxylin-eosin stain, original magnification x100. Direct immunofluorescence was negative.

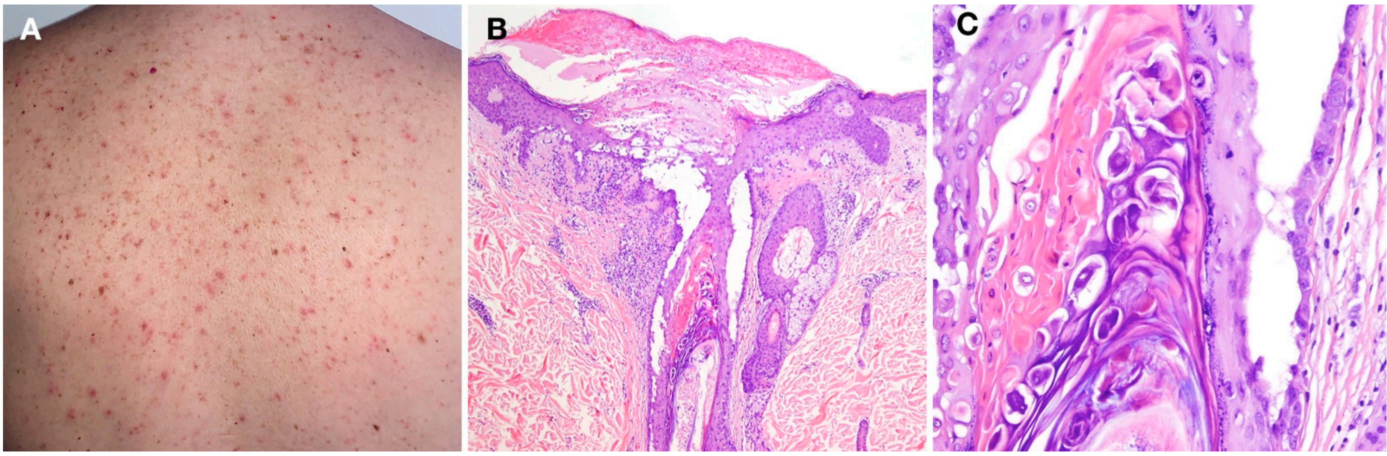

Figure S3. Patient 32 from Supplementary Table 1. (A) 50-year-old woman presented erythematous and purpuric papules on the back; (B) Histology showed an intraepidermal and intrafollicular vesicle in relation with follicular infundibulum, extensive suprabasal acantholysis, and mild superficial perivascular lymphocytic infiltrates (Hematoxylin-eosin stain, original magnification x40). Amplified image shows suprabasal acantholysis with abundant dyskeratotic keratinocytes and corps ronds (Hematoxylin-eosin stain, x100).

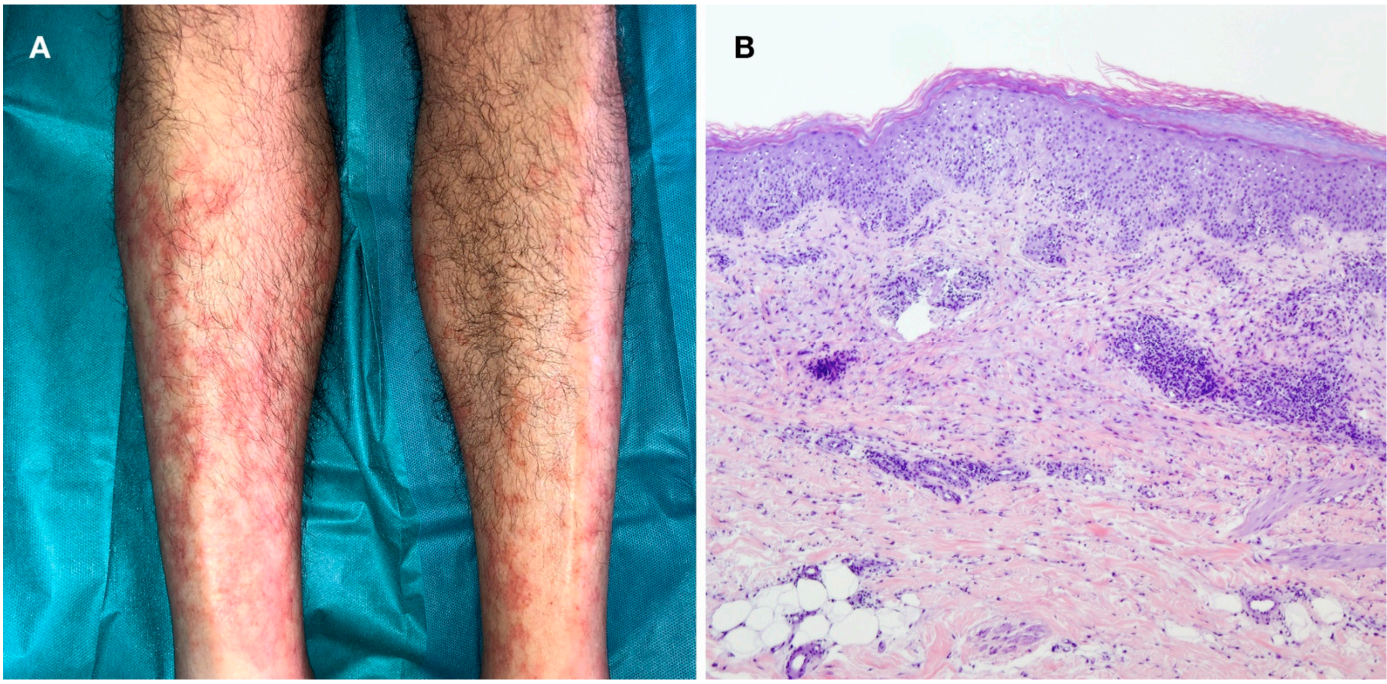

Figure S4. Patient 35 from Supplementary Table 1. (A) 70-year-ol male presented with pruritic erythematous rash on lower limbs with a livedo reticularis pattern. (B) Slight acanthotic epidermis with spongiosis and lymphocytic and eosinophilic exocytosis, and focal vacuolization along the basement membrane. There were moderate lymphocyte and eosinophil perivascular infiltration in the superficial and mid dermis, (Hematoxylin-eosin stain, and original magnification x 40). Direct immunofluorescence was negative.
